# Quantification of epigenetic biomarkers: an evaluation of established and emerging methods for DNA methylation analysis

**DOI:** 10.1186/1471-2164-15-1174

**Published:** 2014-12-23

**Authors:** Nicholas Redshaw, Jim F Huggett, Martin S Taylor, Carole A Foy, Alison S Devonshire

**Affiliations:** LGC, Queens Road, Teddington, Middlesex, TW11 0LY UK; Medical and Developmental Genetics Section, MRC Human Genetics Unit, IGMM, University of Edinburgh, Edinburgh, UK

**Keywords:** DNA methylation, Reference material, Digital PCR, NGS, Quantification

## Abstract

**Background:**

DNA methylation is an important epigenetic mechanism in several human diseases, most notably cancer. The quantitative analysis of DNA methylation patterns has the potential to serve as diagnostic and prognostic biomarkers, however, there is currently a lack of consensus regarding the optimal methodologies to quantify methylation status. To address this issue we compared five analytical methods: (i) MethyLight qPCR, (ii) MethyLight digital PCR (dPCR), methylation-sensitive and -dependent restriction enzyme (MSRE/MDRE) digestion followed by (iii) qPCR or (iv) dPCR, and (v) bisulfite amplicon next generation sequencing (NGS). The techniques were evaluated for linearity, accuracy and precision.

**Results:**

MethyLight qPCR displayed the best linearity across the range of tested samples. Observed methylation measured by MethyLight- and MSRE/MDRE-qPCR and -dPCR were not significantly different to expected values whilst bisulfite amplicon NGS analysis over-estimated methylation content. Bisulfite amplicon NGS showed good precision, whilst the lower precision of qPCR and dPCR analysis precluded discrimination of differences of < 25% in methylation status. A novel dPCR MethyLight assay is also described as a potential method for absolute quantification that simultaneously measures both sense and antisense DNA strands following bisulfite treatment.

**Conclusions:**

Our findings comprise a comprehensive benchmark for the quantitative accuracy of key methods for methylation analysis and demonstrate their applicability to the quantification of circulating tumour DNA biomarkers by using sample concentrations that are representative of typical clinical isolates.

**Electronic supplementary material:**

The online version of this article (doi:10.1186/1471-2164-15-1174) contains supplementary material, which is available to authorized users.

## Background

The methylation of CpG dinucleotides is a common epigenetic mechanism in eukaryotes that plays an essential role in the regulation of gene activity. Defects in methylation have been described in several human diseases, most notably cancer (reviewed in [1]). Alterations in the methylation status of CpG islands in human cancers can lead to genomic instability and silencing of tumour suppressor genes [2–5]. The strong correlation between methylation status and cancer development and progression has led to a growing interest in the use of methylation markers in circulating DNA for cancer diagnosis and prognosis [6, 7]. However, in order for such clinical applications to be developed, further work is required to determine which are the optimal methodologies to quantify methylation status accurately and reproducibly. Indeed, numerous technologies are used for methylation analysis (reviewed in [8] and [9]) and many of these have not previously been evaluated in terms of their quantitative accuracy, precision and repeatability of measurement.

The majority of the most commonly used methods for DNA methylation analysis are those based on sodium bisulfite conversion or methylation-dependent or -sensitive restriction enzymes (defined as MSREs or MDREs respectively throughout). In the first, sodium bisulfite is used to hydrolytically deaminate unmethylated cytosine to uracil while leaving methylated cytosines unchanged [10–12]. Bisulfite converted DNA can be used to assess methylation status by several methods, including PCR and sequencing. Clonal sequencing of bisulfite converted DNA is considered to be the ‘gold standard’ for methylation quantification, as it allows the high-throughput identification of all the methylated cytosines within an extensive region combined with single molecule quantification [13]. The development of pyrosequencing and Next Generation Sequencing (NGS) in recent years has provided an increased genomic coverage and sequencing depth [14–17]. MethyLight [18, 19] is one of the most common PCR based approaches; it involves the bisulfite conversion of DNA followed by real-time quantitative PCR (qPCR) by using primers and hydrolysis probes that are complementary to either the methylated or unmethylated bisulfite-converted DNA sequences. The MethyLight approach has also been applied to a recently developed microfluidic digital PCR platform [20]. Digital PCR (dPCR) involves the distribution of samples over hundreds of reaction wells resulting in each well containing either one or no template molecules, allowing a digital readout of the number of molecules present in the distributed sample, and the absolute quantification of copy number without the need for calibration [21, 22]. An alternative to the sodium bisulfite conversion based methods are those that utilise MSREs and MDREs. One such method involves qPCR amplifying a target region spanning several CpG sites that have been digested by one or more MSREs or MDREs [23–25]. MSREs are unable to digest restriction sites containing methylated cytosine residues, whereas MDREs require the presence of methylated DNA to induce strand cleavage. By comparison of expression levels with a mock digested template, the relative amounts of methylated and unmethylated DNA can be calculated [23, 24]. Recent studies have also applied RE digestion analysis to dPCR platforms [26, 27], with Hindson *et al*. [26] demonstrating a superior precision and sensitivity of measurement compared to qPCR.

In this study, we investigate the suitability of a range of methods based on bisulfite conversion and restriction enzyme digestion for accurate quantification of methylated DNA copies. The methylation status of the p14^ARF^ (alternative reading frame) gene was chosen as the target for quantification, as it is a putative tumour suppressor gene that has been shown to be hypermethylated in human cancers [28–31] and offers potential as a candidate biomarker [32–36]. The comparison study was performed using a panel of standards containing a mixture of methylated and unmethylated DNA combined in a range of known ratios. We analysed quantities of DNA that were representative of clinical samples, such as circulating cell free DNA, which is typically present at 10^3^ genomic copies per mL plasma [37], in order to make this study relevant to the use of methylation biomarkers in non-invasive diagnosis. This material was used to compare restriction enzyme (defined as RE throughout)- and MethyLight-based analysis using qPCR and dPCR platforms and to evaluate bisulfite amplicon NGS. Our findings provide the first comprehensive comparison of these methods and we also develop a novel strategy for absolute quantification of methylated DNA by MethyLight dPCR.

## Results

To assess the accuracy and precision of DNA methylation quantification technologies, five commonly used methods for the quantification of the proportion of methylated DNA molecules were investigated by analysing the methylation status of the biomarker, p14^ARF^, with each method targeting the same region of the p14^ARF^ promoter (Additional file 1), using a panel of DNA standards combined in a range of methylated and unmethylated ratios. Aliquots of a single preparation of this panel were used for analysis by all of the methylation quantification methodologies in a series of independent experiments in order to test the robustness of the approaches.

### RE digestion qPCR and dPCR quantification

To evaluate RE digestion based PCR (termed RE digestion (q/d)PCR hereafter) quantification of DNA methylation, the panel of methylated/unmethylated DNA standards were treated with either MSREs which cleave restriction sites when unmethylated or MDREs which induce strand cleavage only in the presence of methylated DNA, in three independent experiments replicating the whole process. Both classes of restriction enzyme showed a good performance in terms of the efficiency and specificity of digestion. For example, the 0% methylated sample treated with MSREs showed no detection of p14 promoter DNA when measured either by qPCR or dPCR (Table 1). The 100% methylated sample did not show a complete digestion with the MDRE treatment, however only minimal amounts of template were present following digestion compared to controls used for normalisation (Table 1).

**Table 1:** **Restriction enzyme qPCR and dPCR**

Expected methylation (%)	Average methylation ± standard deviation (%)			
	qPCR		dPCR	
	MDRE	MSRE	MDRE	MSRE
100	97 ± 1^¥^	100	94 ± 6^*^	100
90	87 ± 6^+^	85 ± 4	86 ± 6	63 ± 12
75	79 ± 8^‡^	69 ± 17	74 ± 13	93 ± 32
50	42 ± 22^¥,+,‡^	36 ± 11^*,¥,+^	59 ± 16^*^	55 ± 11^*,¥,+^
25	25 ± 9	21 ± 4^*,‡^	31 ± 17	24 ± 7^*,‡^
10	22 ± 20	7 ± 1^¥^	11 ± 28	12 ± 5^¥^
0	0	0^+,‡^	0	0^+,‡^

The average % methylation calculated from three independent qPCR and dPCR measurements of a panel of methylated/unmethylated DNA standards using Methylation-Dependent Restriction Enzyme (MDRE) and Methylation-Sensitive Restriction Enzyme (MSRE) treatments; Each PCR measurement was performed using an independent RE digestion as template. Data normalised to the 100% methylated sample for MSRE assays and 0% for MDRE assays. Statistical comparisons using a One-Way ANOVA were performed on the data where each RE class was used within its optimal template range (0-50% for MSRE; 50-100% for MDRE); all comparisons between samples that were significant at the level of *p* < 0.05 are shown with pairs of the same symbols (*^,¥,+,‡^) denoting which two samples were compared, e.g. for MDRE dPCR, only the 50% and 100% methylated (expected % methylation) data points were significantly different from each other at the level of *p* < 0.05. Data points that were outside the viable range of the assay (< 0% or > 100% methylation) were removed from the analysis; all experimental conditions were *n* = 3 with the exception of the following that were *n* = 2: 10% and 25% MDRE qPCR and 90% MSRE qPCR (expected % methylation).

**Figure 1:**
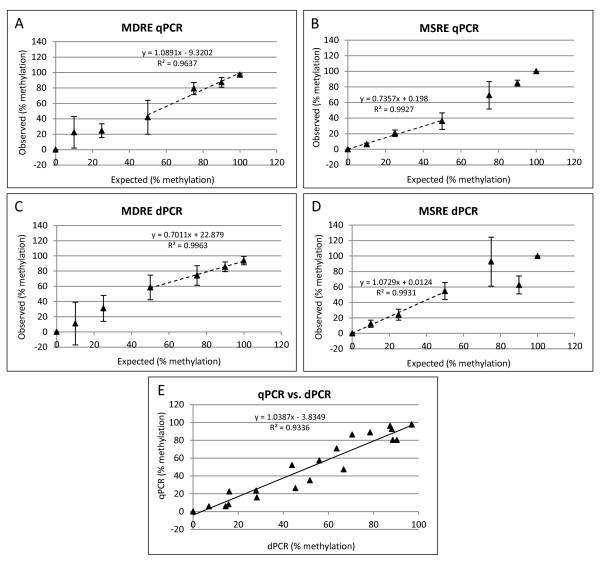
**Restriction enzyme qPCR and dPCR.** Correlation between expected and observed percent methylation for Methylation Dependent Restriction Enzyme (MDRE) **(A,C)** and Methylation Sensitive Restriction Enzyme (MSRE) **(B,D)** qPCR **(A,B)** and dPCR **(C,D)** analysis. Correlation performed with samples which comprise the optimal working range of the respective enzyme classes: 0-50% for MSRE **(B,D)** and 50-100% for MDRE **(A,C)** (dotted lines). Data points that were outside the viable range of the assay (< 0% or > 100% methylation) were removed from the analysis. All experimental conditions were *n* = 3 with the exception of the following which were *n* = 2: 10% and 25% MDRE qPCR and 90% MSRE qPCR (expected % methylation). Error bars show ± Standard Deviation of three independent replicate measurements. **(E)** Correlation between RE qPCR *vs*. dPCR measurements using the data points in which the restriction enzymes are within their optimal working range. A single outlying data point was removed from the correlation analysis. All correlations were significant at *p* < 0.0001 except for expected *vs*. MDRE dPCR which was *p* = 0.0011.

The average percent methylation measured in all reactions is presented in Table 1 and Figure 1 (see Materials and Methods for details of percent methylation calculations). Consistent with a previous study [23], the data demonstrate how the variability of measurement can increase when the REs are used within a range of template % methylation that is not optimal for accurate PCR quantification, e.g. standard deviation measurements are generally higher in the 0-25% methylated template range with the MDRE digestion (Table 1, Figure 1A,C). Furthermore, outside the optimal template range, several data points displayed values that were outside the feasible range of the assay (< 0% or > 100% methylation) and therefore were removed from the analysis (Table 1 and Figure 1A,B). Therefore, in order to assess the accuracy of this method, the correlation between expected and observed percent methylation for the data points was analysed where each class of RE was in its optimal range (0-50% methylated data points for MDRE and 50-100% methylated for MSRE, Figure 1A-D). Figure 1 demonstrates that, within the optimal template methylation ranges, both MS- and MDREs show a significant correlation with the expected percent methylation for both qPCR and dPCR measurements (*p* = 0.0011 for MDRE dPCR, all others are *p* < 0.0001) however when data from the entire range are included, the correlation between observed and expected values generally decreases (data not shown), highlighting the importance of using the digestion enzymes within their optimal working range.

The correlation between the qPCR and dPCR measurements, using the optimal template methylation range values, was also significant (R^2^ = 0.93; *p* < 0.0001; Figure 1E) however, analysis of the standard deviation values (Table 1 and Figure 1) revealed that, in the majority of cases, dPCR showed greater variability of measurement between replicates compared to qPCR. Precision of measurement was tested by performing a one-way ANOVA statistical analysis of the data to determine which observed differences in methylation status were statistically significant. This analysis revealed that the RE qPCR approach could accurately discriminate differences in ≥ 25% methylation, however comparisons of 10% and 15% differences could not be resolved, indicating that the precision of this method was not accurate enough to use for differences of < 25% methylation. For RE dPCR, the MSRE approach showed a comparable performance to qPCR, with the same comparisons between samples showing statistically significant differences. However, the MDRE dPCR approach showed poorer precision than MDRE qPCR, with only the 50% *vs*. 100% methylation comparison showing a statistically significant difference (Table 1).

### MethyLight qPCR and dPCR analysis

**Figure 2:**
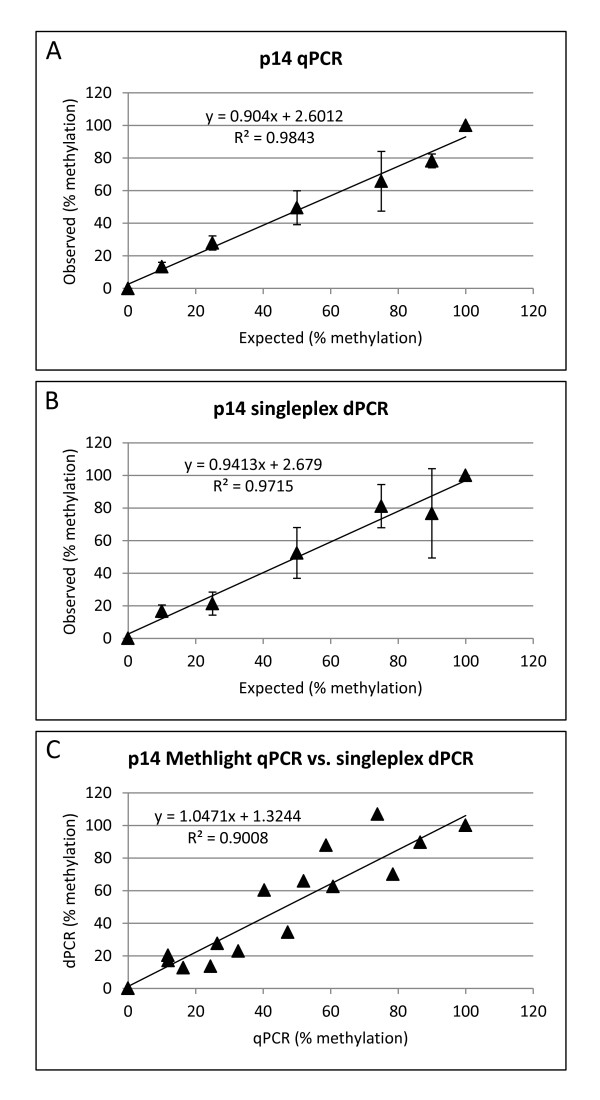
**MethyLight qPCR and singleplex dPCR.** Correlation between expected and observed % methylation for MethyLight qPCR **(A)** and singleplex dPCR **(B)** using the p14_M assay. Error bars show ± Standard Deviation of three independent replicate measurements. **(C)** Correlation between MethyLight qPCR *vs*. singleplex dPCR. All correlations were significant at *p* < 0.0001.

To analyse and estimate the variation of quantification of methylation by MethyLight qPCR and dPCR, three independent bisulfite conversions were performed on the panel of methylated/unmethylated DNA. The replicates of bisulfite converted DNA were quantified by qPCR and dPCR using the same hydrolysis probe assay. Figures 2A and B show that there was a strong correlation between the average observed and expected percent methylation when measured by qPCR and dPCR across the full range of methylation (*p* < 0.0001 for all correlations). The p14 assays showed a good specificity of measurement for the methylated template with both MethyLight qPCR and dPCR, as the 0% methylated template was not detected by either method with the p14_M assay.

However, the performance of qPCR and dPCR in terms of reproducibility of measurement revealed that, in the majority of cases, the standard deviation measurements were higher for dPCR compared to qPCR (Table 2). The inferior precision of MethyLight dPCR was also demonstrated in that qPCR was able to resolve differences in percent methylation of ≥ 25% however none of the statistical comparisons below a 50% difference in methylation were significant for MethyLight dPCR using the p14_M assay in singleplex (Table 2).

**Table 2:** **Methylight qPCR and dPCR**

Expected methylation (%)	Average methylation ± standard deviation (%)				
	qPCR	dPCR			
	P14_M	P14_M	P14_M	P14_M2	P14_M + P14_M2
	Singleplex	Singleplex	Duplex	Duplex	Duplex
100	100^*^	100	100	100	100
90	78 ± 4^¥^	77 ± 27	99 ± 19^*^	86 ± 20	92 ± 19
75	66 ± 18^*^	81 ± 13	86 ± 20	77 ± 17	81 ± 19
50	49 ± 10^¥,+^	52 ± 16	58 ± 10^*,¥^	57 ± 28^*^	56 ± 18^*^
25	28 ± 4^‡^	21 ± 7	31 ± 5	28 ± 2	29 ± 4
10	13 ± 3^+^	17 ± 4	18 ± 6^¥^	9 ± 15^*^	13 ± 10^*^
0	0^‡^	0	0	0	0

The average % methylation calculated from three independent qPCR and dPCR measurements of three independent bisulfite conversions of a panel of methylated/unmethylated DNA standards; Data for dPCR show measurements from the P14_M assay used in singleplex and for the P14_M and P14_M2 assays used together in duplex, showing the % methylation for each assay when analysed individually and with the estimated targets combined (P14_M + P14_M2). Symbols denote statistical comparisons using a One-Way ANOVA test. All comparisons between samples with expected differences in methylation of ≥ 50% were significant at the level of *p* < 0.05 (with the exception of 50% *vs*. 100% methylation with MethyLight P14_M2 Duplex) and are not shown; all comparisons between samples of differences between 0-40% expected methylation that are significant (*p* < 0.05) are shown with pairs of the same symbols (*^,¥,+,‡^) denoting which two samples were compared, e.g. for dPCR P14_M2 duplex, only the 10% and 50% methylated (expected % methylation) data points were significantly different from each other at the level of *p* < 0.05.

### Duplex MethyLight dPCR analysis

A dPCR approach offers the possibility of absolute quantification of methylated DNA without a calibration curve [21]. However one source of uncertainty with respect to the accuracy of copy number concentration measurements is whether the DNA template is present in single-stranded or double-stranded conformation [38]. In the former case individual strands of the duplex can become separated into different partitions and be amplified, resulting in a ‘double-count’ which could lead to overestimation of template concentration under the assumption of double-stranded conformation [38]. We hypothesised that this source of uncertainty could be resolved for dPCR analysis of methylated DNA as following bisulfite treatment the DNA strands are no longer complementary, enabling each strand to be discriminated [13]. In the case of the region targeted in this study, 47% of nucleotides within the MethyLight p14 assay target region are non-complementary after bisulfite conversion of the 100% methylated template and 68% for the 0% methylated template.

**Figure 3:**
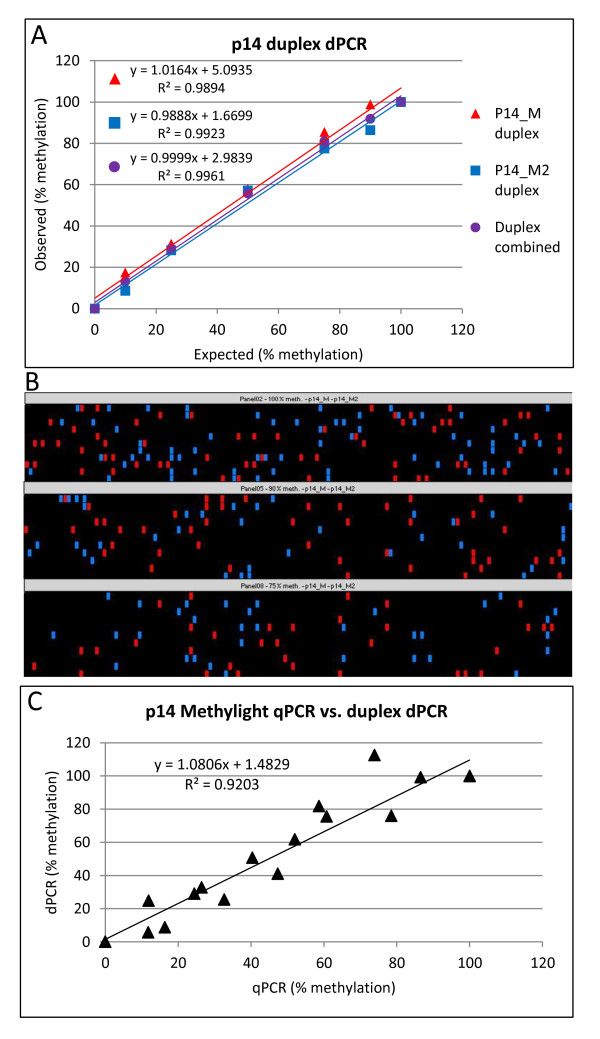
**MethyLight duplex dPCR. (A)** Duplex p14 dPCR assay showing data for p14_M and p14_M2 assays separately and with estimated targets for both assays combined. **(B)** dPCR heatmap showing distribution of p14_M (red) and p14_M2 (blue) positive chambers in a duplex reaction showing three example panels of a dPCR plate. **(C)** Correlation between MethyLight qPCR *vs*. duplex dPCR (estimated targets for both assays combined). All correlations were significant at *p* < 0.0001.

An additional MethyLight assay (‘P14_M2’) was designed (Additional file 2) to amplify the opposite of the two resultant heteroduplex strands to that targeted by the P14_M assay (previous section). To enable the assays to be distinguished, the probe for each assay was labelled with a different fluorophore, allowing duplexing of the reaction. The p14_M2 assay also showed high specificity for methylated P14 DNA and did not amplify non-methylated templates (Figure 3).

Figure 3A shows the observed *vs*. expected percent methylation for the dPCR analysis showing the p14_M and p14_M2 primers and probe when used in duplex (data shown for each assay individually and when estimated targets from both assays are pooled). The data demonstrate that both single- and duplex dPCR assays show a strong correlation with the expected values (*p* < 0.0001).

The duplex approach to MethyLight dPCR demonstrates that amplifications of the two non-complementary DNA strands (by p14_M and p14_M2 assays) are independently localised, as seen in the dPCR heat maps in three example panels (Figure 3B) (< 0.02% of positive chambers showed amplification with both assays on average over all panels of all experiments). This confirms that the starting template is largely in a single-stranded conformation and that each assay is specific to either strand of the starting template as non-specific amplification of the ‘non-template’ strand would have resulted in amplification of both assays in a large number of the chambers. Similarly to the singleplex MethyLight dPCR (Figure 2C), there was a significant correlation between the qPCR and duplex dPCR measurements (Figure 3C; *p* < 0.0001).

### Absolute quantification of RE digested- and bisulfite-treated DNA

**Figure 4:**
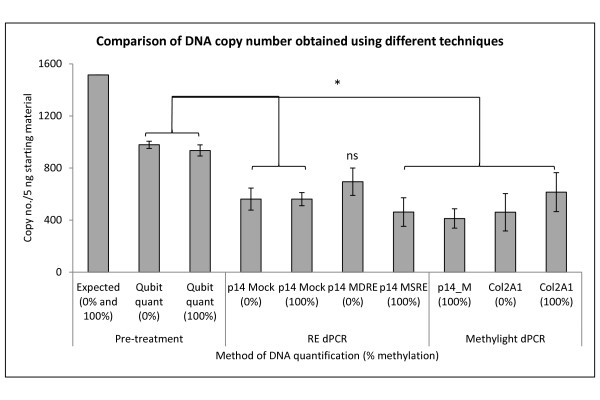
**Comparison of DNA copy numbers obtained using different techniques.** DNA copy numbers of methylated/unmethylated DNA standards based on specifications of manufacturer (Expected), measured by flourometer and of p14 by Restriction Enzyme (RE) dPCR and p14 and COL2A1 with MethyLight dPCR in the 0% and 100% methylated samples. Copy numbers shown were obtained from 5 ng starting material (based on expected DNA quantity), pre-bisulfite conversion and RE digestion. RE dPCR data shows p14 copy number from the mock, MSRE and MDRE treatments. MethyLight dPCR data shows copy number obtained using the p14_M assay in singleplex and the methylation independent control COL2A1. Statistical comparisons are for Student’s t-test (* = *p* < 0.05; ns = not significant). Error bars show ± Standard Deviation of measurement.

In order to assess the comparative yield between the bisulfite and RE approaches, the copy number obtained from RE dPCR and MethyLight dPCR was compared (Figure 4). The DNA quantified in our experiments before RE digestion or bisulfite treatment was ~ 1500 genomic copies based on the manufacturer’s specifications of the 100% methylated and unmethylated DNA standards. However, our fluorometric measurements (Qubit) indicated the copy number to be ~ 950 genomic copies (Figure 4). The number of amplifiable copies of the p14 and COL2A1 genomic regions detected by MSRE-dPCR, mock digestion conditions and MethyLight dPCR was less than the values based on Qubit analysis (*p* < 0.05) with the exception of the MDRE dPCR assay (0% methylated template (*p* = not significant)). No significant differences were observed between MSRE or MDRE analysis of their control templates (100% and 0% methylated respectively) and mock digest conditions. Likewise, comparison of the copy numbers of p14 obtained by MethyLight dPCR with those obtained for the methylation independent control, COL2A1, in the same sample (100% methylated), did not reveal any significant differences (*p* > 0.05, student’s *t*-test), indicating a comparable efficiency for the p14 and COL2A1 assays, confirming that COL2A1 was a suitable reference for the effects of sample processing on the target gene of interest.

### Bisulfite amplicon NGS

**Figure 5:**
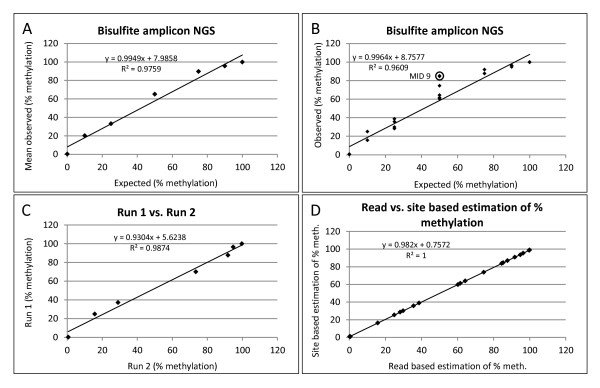
**Bisulfite Amplicon NGS.** Analysis showing read-based estimates of percent methylation **(A-C). (A,B)** Correlation between expected and observed percent methylation showing **(A)** average percent methylation for all samples across both amplicon NGS experiments or **(B)** all data points plotted individually excluding Multiplex Identifier (MID)9 samples from the analysis in A and B. **(C)** Correlation between two bisulfite amplicon NGS experiments (including MID9). **(D)** Correlation between read- and site-based estimates of percent methylation. All correlations were significant at *p* < 0.0001.

To assess bisulfite amplicon NGS, sequencing experiments (*n* = 2 replicating the bisulfite, PCR and sequencing protocols) were performed using the Roche 454 GS Junior amplicon sequencing protocol on independent bisulfite conversions of the panel of methylated/unmethylated DNA. In order to quantify multiple samples in a single experiment, template DNA was PCR amplified using fusion primers containing MIDs. The assignment of MIDs to samples was randomised across the two experiments (Table 3). Two of the samples (25% and 50% methylated) were replicated within each experiment using different MID fusion primers in order to assess intra-run measurement repeatability. Figure 5A and B show strong correlations (*p* < 0.0001) between the expected and observed percent methylation values, both when averaging the observed percent methylation for all samples across both experiments (Figure 5A; R^2^ = 0.976) or when performing the correlation with individual data points (Figure 5B; R^2^ = 0.961). Although there was a strong correlation with the expected values, linear regression showed a clear trend for the bisulfite amplicon NGS to over-estimate the percent methylation (intercept of + 8%, Figure 5A,B), evident in sample of mixed ratios by 1.32-fold on average. This effect was not observed in the 0% and 100% samples with less than 1% of reads not matching the expected sequence, resulting in non-random distribution of the residuals of the linear regression analysis (Additional file 3). The over-estimation bias had a greater impact for the lower percent methylation samples; for example, 10% methylation was over-estimated by 2.03-fold, whereas 90% methylation was over-estimated by 1.06-fold (Figure 5, Table 3).

**Table 3:** **MethyLight qPCR and dPCR**

Run no.	MID no.	% methylation	
		Expected (× replicate no.)	Observed
1	1	100	100
	2	90	96.4
	3	75	87.7
	9	50 × 1	85.3
	*4*	50 × 2	60.2
	7	50 × 3	64.3
	5	25 × 1	35.6
	10	25 × 2	38.7
	8	10	24.9
	6	0	0.2
2	7	100	99.7
	3	90	94.8
	8	75	91.8
	9	50 × 1	84.5
	10	50 × 2	61.5
	6	50 × 3	74.5
	1	25 × 1	29.8
	4	25 × 2	28.2
	5	10	15.7
	2	0	0.6

The % methylation determined by bisulfite amplicon NGS using Multiplex Identifier (MID) fusion primers to amplify samples from a panel of methylated/unmethylated DNA standards mixed in a range of known ratios. The 25% and 50% methylated samples were replicated (× 1-3) within each of two runs.

The correlation between experiments 1 and 2 was strong (Figure 5C; R^2^ = 0.987; *p* < 0.0001) suggesting a high level of reproducibility of the whole process. When comparing results from replicate samples with different MID fusion primers, however, one of the MID fusion primers (MID9) caused a clear bias in both experiments (Figure 5B) and therefore was removed from the correlation analysis (Figure 5A and B) (*t*-test showed that, for the 50% methylated sample, the MID9 values were significantly higher at *p* < 0.05 than the values excluding MID9). With the exception of the MID9-tagged amplicon, the difference in percent methylation between replicates (*n* = 2) ranged from 1% to 13%. The range of differences between replicates were similar when comparing within and between the two sequencing experiments, indicating that sources of variation within an experiment (e.g. different MID primers, independent PCRs) were the major sources of variability compared to factors differing between experiments (e.g. bisulfite treatment, library preparation and sequencing). Figure 5D shows that there was a very strong correlation between the read- and site-based estimations of methylation indicating no measurable effect of PCR chimerism.

## Discussion

DNA methylation status has been proposed as a biomarker for many diseases including cancer [6, 7]. However, for the quantification of DNA methylation to be translated to clinical care, further work is needed to determine the accuracy of the available methods. In this study five methods were compared, comprising of two strategies to distinguish between methylated and unmethylated DNA (RE digestion or bisulfite conversion), and three analytical platforms (qPCR, dPCR and NGS).

### Accuracy and precision of qPCR-based methods

Of the qPCR methods tested, MethyLight qPCR demonstrated superior accuracy as reflected in good linearity over a wider range of % methylation compared to MSRE- or MDRE-based analyses which were optimal within the 0-50% and 50-100% methylation ranges respectively. This highlights the importance that both digestion strategies are applied when using this technique on samples of an unknown methylation status. However, MethyLight was also less accurate above 75% methylation. The MSRE/MDRE qPCR findings are consistent with Oakes *et al*. [23] which also identified a decreased accuracy of measurement in > 75% methylated samples for MSRE and < 25% for MDRE. This is likely due to difficulties in accurately discriminating small differences between the sample and control (100% methylated DNA for MethyLight and MSRE qPCR and 0% methylated DNA for MDRE qPCR) by PCR, i.e. for RE qPCR analysis, a 10% difference translates to a difference in Cq of only 0.2, which is comparable to the typical standard deviation of Cq technical replicates. In addition, RE digestion and bisulfite conversion are sources of variability as the initial treatment of DNA can cause non-specific template degradation and confound the relative comparison between samples and control 100% methylated DNA [39].

The precision of both MethyLight- and RE-qPCR approaches was limiting in terms of discrimination between samples with small differences in methylation content. Our experiments demonstrated that the precision of MethyLight measurements was sufficient to be able to discriminate differences of ≥ 25% methylation but not 10% or 15%, which is comparable with a previous study by Ogino *et al*. (2006) [40]. Both MethyLight qPCR assay and bisulfite conversion were observed to contribute to overall technical error [40]. The precision of RE qPCR, within the optimal ranges of the restriction enzymes, was comparable to MethyLight qPCR, as statistical analysis revealed that differences in methylation of ≥ 25% could be reliably discriminated but not below this.

Hashimoto *et al*. [24] reported that when using a MSRE qPCR approach with four independent replicate experiments, differences between two samples of > 11% in methylation status could be determined, therefore increased replication may improve precision. Holemon *et al*. [25] found, when using a combined MDRE and MSRE qPCR approach, that the standard deviation measurements of replicate qPCR experiments were greater than that of replicate RE digestions. This further highlights how the number of replicate assays is as important a consideration when determining the confidence or uncertainty in the measurement, as both sample treatments (RE digestion or bisulfite conversion) and analysis stages contribute to the overall measurement variability. It is crucial that both stages are replicated in order to estimate the precision of measurements accurately. Control samples of defined methylation content such as those tested in this study may be a useful means of monitoring whole process precision.

### Application of dPCR to quantification of methylation

MethyLight has recently been applied to a dPCR platform [20], however its use for methylation quantification has not been extensively investigated; this study provides the first direct comparison of MethyLight qPCR with MethyLight dPCR and RE qPCR with RE dPCR. The correlation between qPCR and dPCR measurements for both upstream treatments was high (R^2^ ≥ 0.90), suggesting similar linearity to qPCR approaches. However, the precision of both MethyLight and MSRE/MDRE-dPCR was found to be worse compared to the qPCR measurements. This is likely to be as a result of using a relatively low concentration of template (λ < 0.2 for all samples, Additional file 4) in order to be representative of typical clinical samples as our experiments were focused on the application of methylation quantification to biomarker analysis. Based on Poisson statistics, dPCR is predicted to demonstrate highest precision at lambda values of between 1.0 and 2.0 [41]. Indeed, Hayden *et al*. [42] observed that dPCR showed higher measurement variability compared to qPCR when measuring human cytomegalovirus DNA in low concentration clinical samples. As in the Hayden *et al*. [42] report, the quantity of DNA analysed in our study by dPCR within the partitions of the IFC (0.65 ng) was lower than that analysed per qPCR reaction (5 ng). dPCR platforms with higher sample volume input and a greater number of partitions compared to the BioMark (such as the Bio-Rad Droplet Digital System and Life Technologies QuantStudio 3D Digital PCR System) may improve MethyLight- or RE-based dPCR precision.

### Absolute quantification of methylated DNA templates

Our study extended the application of dPCR to analysis of DNA methylation by demonstrating how dPCR can be useful for studying the impact of upstream treatments on the number of amplifiable copies in a sample. Our dPCR experiments showed comparable p14 DNA copy numbers in bisulfite-treated samples with those treated with REs or mock conditions. A previous study using an earlier bisulfite conversion protocol [39] demonstrated a loss of 84-96% of DNA after bisulfite treatment. Our study demonstrates that more recently developed protocols that use less harsh treatment conditions, may reduce sample loss significantly. However both MethyLight and RE-based measurements were between 1.5- and 2.3-fold lower than the copy number predicted by fluorometric quantification, suggesting that additional sources of bias may reduce the number of amplifiable copies.

We also developed a novel strategy for absolute quantification of methylated DNA by measuring both strands of the double helix post bisulfite-treatment. This method addresses an important source of uncertainty for dPCR-based quantification related to whether the template DNA is in single-stranded or double-stranded form [38]. Our data showing the location of the two strands of the double helix into separate partitions confirms the single-stranded nature of the bisulfite converted DNA [13]. By quantifying all of the template strands in a reaction, this method provides an improvement for the absolute quantification of DNA copy number which could be particularly useful for the assignment of values to reference materials [38].

### Accuracy and precision of bisulfite amplicon NGS method

Bisulfite amplicon NGS using 454 pyrosequencing has been evaluated previously in analyses of clinical samples but has not been assessed in terms of its accuracy of measurement or directly compared against other technologies. The bisulfite amplicon NGS approach using the Roche Junior platform demonstrated superior precision compared to qPCR/dPCR methods, as evidenced by the high concordance of replicate experiments (R^2^ = 0.987). However the accuracy of bisulfite amplicon NGS was inferior to that of qPCR and dPCR as demonstrated by a systematic bias to over-estimate the percent methylation, with every observed value being above the expected, with the exception of the 0% and 100% methylated samples. On average, the measured values were 1.32-fold above the expected. It cannot be ruled out that the bias resulted from initial inaccuracy in template quantification and/or preparation prior to bisulfite amplicon NGS analysis. However, the same material was used for analysis by all of the other methodologies, none of which also showed a systematic over-estimation of methylation suggesting the bias was specific to this procedure.

Our findings are also consistent with a previous study which found a pyrosequencing-based method to have a far superior precision of measurement compared to MethyLight but it also demonstrated a systematic over-estimation in percent methylation [43]. However interrogation of passed and failed reads from the GS Junior sequencer revealed that these did not differ between the unmethylated *vs*. methylated sequences, suggesting that the bias did not stem from differences in the number of homopolymeric tracts in the sequences which may cause errors in pyrosequencing data due to the incorporation of multiple nucleotides in one flow [44].

It is more likely that the methylation bias was at the level of PCR as several previous studies have shown how bisulfite PCR, using methylation independent primers, can exhibit a strong methylation bias [45–47]. In these cases, however, the PCR bias results in an underestimation of methylation levels which was attributed to methylated DNA containing secondary structures in the template associated with a reduction in PCR efficiency compared to unmethylated sequences [45]. Other studies have observed a PCR-related bias towards methylated DNA: Dabney and Meyer found that certain DNA polymerases favour template molecules of higher GC content [48] while Shen *et al*. found that higher annealing temperatures increased the estimation of methylation content in samples of low to medium methylation (20-50%) [49], suggesting that changes in the choice of enzyme and/or annealing temperature may improve the bias observed in this study.

In addition, our study demonstrates that there can be an interaction between sample identifier primer tags (MIDs) and PCR bias. One of the MID-tagged primers (MID9) showed methylation values that were clear outliers in both sequencing experiments: measurements of 85% were observed for the 50% methylated sample compared to an average measurement of 65.1% for other MID tags (Figure 5 and Table 3). A previous bacterial metagenomics study demonstrated that barcoded primers can introduce biases in PCR that translate into less reproducible data sets and can reduce apparent bacterial diversity, due to preferential amplification of certain 16S rRNA sequences [50]. This is also important for DNA methylation analysis as multiplexed amplicon sequencing is being applied to large-scale analysis of multiple methylation biomarkers [16, 51]. In summary, further validation of the quantitative accuracy of deep sequencing is warranted, reinforcing the importance of reference controls to identify any biases that may be introduced.

## Conclusions

Although previous investigations have discussed the relative merits of bisulfite conversion *vs*. RE digestion based methods of methylation quantification [18, 24, 25], this is the first study to directly compare both upstream treatments with alternative downstream PCR methods and platforms. Whilst MethyLight assays demonstrated an extended quantitative range compared to MSRE-or MDRE-qPCR, MethyLight is a methylation specific PCR approach and is therefore limited to the CpGs that lie within the region that the primers and probes are designed to bind. In contrast, REs can be used that will target a larger number of methylation sites within a target region. Bisulfite amplicon NGS has the advantage over the other tested methods that it provides information on the methylation status of all CpG residues in an amplicon of the gene promoter that is larger than that of typical qPCR/dPCR assays; this technique displayed superior measurement precision compared to the others evaluated, however a systematic bias to over-estimate methylation rate was evident. These findings provide an important benchmark for these methods that assists researchers embarking on methylation studies to determine which method is most suitable for the purposes of their experiments and guide the aspects of these techniques that need to be addressed through the implementation of reference standards for methylation measurements.

## Methods

### Preparation of a panel of methylated/unmethylated DNA standards

A panel of seven DNA standards was prepared by combining the following proportions of methylated and non-methylated Human DNA (Zymo Research, Irvine, CA, USA): 100%, 90%, 75%, 50%, 25%, 10%, 0% methylated in 1.5 mL tubes at a final concentration of 20 ng/μL. The commercially available Human DNA did not require approval by a Research Ethics Committee. DNA was diluted in TE buffer (10 mM Tris pH 8.0, 0.1 mM EDTA) (USB Corporation, Cleveland, OH, USA) and stored at -20°C. DNA was quantified using the Qubit 2.0 Fluorometer with the dsDNA BR Assay (Life Technologies, Carlsbad, CA, USA).

### RE digestion

Three replicate RE digestions were performed on aliquots of a single preparation of the panel of methylated/unmethylated DNA standards. To encourage complete cleavage of the template, double digestions were performed for both the MS- and MDRE digestion reactions. For the MSRE reactions, AciI and HhaI were used, which contain a total of five potential cleavage sites in the p14 target region. For MDRE digestion, FspEI, which recognises seven potential methylated restriction sites, was used in combination with McrBC which cuts at multiple positions in methylated CpG rich sequences between two half-sites of the form (G/A)^m^C at an optimal distance of 55–103 bp. For each replicate, 3 × 10 μL RE digestion reactions were prepared for each sample: MSRE, MDRE and a mock digestion. All reaction components for the digestions were supplied by New England Biolabs (Ipswich, MA, USA). MSRE reactions contained: 1 × Buffer 3, 1 × Bovine Serum Albumin (BSA), 5 U AciI, 5 U HhaI and 100 ng DNA. MDRE reactions contained: 1 × Buffer 4, 1 × BSA, 1 × Enzyme Activator Solution, 1 × GTP, 5 U FspEI, 5 U McrBC and 100 ng DNA. Mock reactions contained 1 × Buffer 4 and 1 × BSA. Reactions were incubated at 37°C for 1 h and subsequently heat inactivated for 20 min at 65°C. Digests were diluted 1 in 10 with TE buffer (10 mM Tris pH 8.0, 0.1 mM EDTA) (USB Corporation) to 1 ng/μL for subsequent analysis and stored at -20°C.

### Sodium bisulfite treatment

Three replicate sodium bisulfite conversion reactions were performed on the panel of methylated/unmethylated DNA standards using the EpiTect Plus Bisulfite Kit (Qiagen, Hilden, Germany) according to the manufacturer’s instructions with 200 ng DNA and ethanol from Sigma (St. Louis, MO, USA). DNA was eluted from DNA spin columns in 15 μL and the volume made up to 50 μL with TE buffer (10 mM Tris pH 8.0, 0.1 mM EDTA) (USB Corporation). Reactions were performed on a GeneAmp 9700 thermocycler (Life Technologies). Samples were stored at -80°C.

### qPCR

qPCR experiments (RE digestion qPCR and MethyLight qPCR) were performed in accordance with the Minimum Information for Publication of Quantitative Real-Time PCR Experiments (MIQE) guidelines (Additional file 5). Reactions were performed using 1 × Taqman Universal PCR mastermix, Cat no. 4304437 (Life Technologies) for RE digestion qPCR and 1 × Taqman Universal PCR mastermix without Uracil-DNA Glycosylase, Cat no. 4324018 (Life Technologies) for MethyLight qPCR in a final volume of 20 μL. All reactions also contained 900 nM final concentration forward and reverse primers, 200 nM Probes (see Additional file 6 for primer and probe sequences and Additional file 7 for details of assay performance) and 5 ng template DNA. All primers and probes were supplied by Sigma (UK). For RE qPCR, reactions contained 5 μL template DNA and MethyLight qPCR contained 1.25 μL DNA. Reactions were performed using a Prism 7900HT Real Time PCR system (Life Technologies). qPCR thermal cycling conditions were as follows: 95°C for 10 min, followed by 40 cycles of 95°C for 15 s, 60°C for 1 min.

For RE digestion qPCR, all reactions were performed in triplicate. At least one PCR NTC was run for each assay as controls on all plates. The SDS software v2.4 (Life Technologies) was used to calculate the quantification cycle (Cq) value. For the RE qPCR assays, percent methylation was calculated by subtracting the mean Cq values of the MSRE or MDRE digested templates from the corresponding mock digest (∆Cq). ∆∆Cq was then calculated by subtracting the ∆Cq of the 100% methylated sample (MSRE digests) or the 0% (MDRE digests) from ∆Cq of the sample in question, and the equations 100 × (2^∆∆Ct^) and 100 × (1-(2^∆∆Ct^)) were used to calculate % methylation respectively [52].

For MethyLight qPCR, quantification was performed using the standard curve method with Bisulfite Converted Methylated Human DNA from Zymo Research. The standard curves consisted of 5 × 1 in 5 dilutions, the highest copy number being 7575 estimated haploid genome copies/reaction. For MethyLight qPCR, standard curves were performed using triplicate measurements for each dilution and duplicates for the panel of methylated/unmethylated DNA standards. For MethyLight qPCR, DNA copy number values were interpolated from standard curves and values for p14 were normalised to methylation independent COL2A1. Normalised copy numbers were then further normalised to the 100% methylated sample.

### Microfluidic dPCR

Microfluidic dPCR experiments (MethyLight dPCR and RE digestion dPCR) were performed in accordance with the Minimum Information for Publication of Digital Quantitative PCR Experiments (digital MIQE) guidelines (Additional file 8) using the Biomark system with 48 panel “qdPCR^TM^ 37 K” integrated fluidic circuits (IFCs), Cat. No. 100–6152 (Fluidigm, South San Francisco, CA, USA). Assays were performed using 1 × Taqman Universal PCR mastermix, Cat no. 4304437 (Life Technologies) for RE digestion dPCR and 1 × Taqman Universal PCR mastermix (no Uracil-DNA Glycosylase), Cat no. 4324018 (Life Technologies) for MethyLight dPCR. All reactions also contained 2 × DA sample loading reagent, 900 nM final concentration forward and reverse primers, 200 nM Probes (see Additional file 2 for details of primer and probe design, Additional file 6 for primer and probe sequences, and Additional file 9 for assay performance) and 5 ng DNA. All primers and probes were supplied by Sigma (UK). For RE dPCR, 20 μL reactions containing 5 μL DNA were prepared and loaded across four panel inlets (5 μL per inlet) with the number of positive amplifications (counts) from the four panels being pooled together. For MethyLight dPCR, 5 μL reactions containing 1.25 μL template DNA were loaded onto each panel inlet. dPCR thermal cycling conditions were as follows: 95°C for 10 min, followed by 45 cycles of 95°C for 15 s, 60°C for 1 min. PCR NTCs were run as controls on all plates. The Fluidigm BioMark Data Collection software (version 4.0.1) was used to analyse the data. The number of positive amplifications (counts) was used for analysis of Cq variation, whereas the number of estimated targets (based on a Poisson correction of the proportion of positive partitions, to estimate the number of copies) was used for quantification of target copy number [53]. The average number of estimated molecules per chamber (lambda, λ) was calculated using the following equation: *λ* = - *ln*(1 - *k*/*n*) where *k* is the number of counts and *n* is the total number of partitions [54]. To calculate percent methylation for the RE digestion dPCR, estimated targets for the digestion reactions were normalised to those of the corresponding mock digest and further normalised to the 100% methylated sample for MSRE and 0% for the MDRE reactions to obtain %RQ. For the MDRE reactions, the final values were calculated using the equation 1 -%RQ. For MethyLight dPCR, estimated targets for p14 assays were normalised to those of the methylation independent COL2A1 assay. Normalised copy numbers were then further normalised to the 100% methylated sample.

### Bisulfite amplicon next generation sequencing

Two replicate sample libraries were prepared from two independent bisulfite conversions and PCR amplifications of the methylated/unmethylated DNA standards (two of the three replicate bisulfite conversions that were also used for MethLight analysis) according to the standard Roche (Basel, Switzerland) GS Junior 454 protocol for amplicon sequencing (January 2013 version) using fusion primers where both the forward and reverse primers contain the Roche adapter sequences and the forward primers also contain a Multiplex Identifier (MID) to enable sequencing reads to be assigned to each sample (see Additional file 6 for fusion primer sequences). Two of the samples (25% and 50% methylated) were replicated in each library preparation (25%, *n* = 2; 50%, *n* = 3) using different MID fusion primers. The assignment of MID fusion primers to samples was randomised in each library preparation (Table 3). Amplicon libraries were generated using the Roche FastStart High Fidelity PCR System according to the manufacturer’s protocol using 20 ng template DNA and the following cycling parameters: 94°C for 3 min, 35 cycles of 94°C for 15 s, 60°C for 45 s, 72°C for 60 s followed by an incubation at 72°C for 8 min. After purification, libraries were quantified using the Qubit 2.0 Fluorometer (Life Technologies) to estimate DNA copy number and analysed with the Agilent Bioanalyzer 2100 to verify product size. Amplicon libraries were combined in an equimolar ratio, using 1 × 10^7^ DNA molecules with 5 × 10^6^ beads (2 to 1 ratio) for emulsion PCR. Two sequencing experiments were performed on the replicate library preparations using the Roche GS Junior Titanium emulsion PCR (Lib-A), Sequencing and PicoTiterPlate Kits according to the manufacturer’s protocols on a Roche GS Junior sequencer.

### Sequencing data analysis

Sequence reads were aligned (Exonerate, version 2.2.0, with options model = affine:local and subopt = F, score = 600) to a 205 nucleotide template p14 reference sequence in which all cytosine residues were masked. Masking avoided bias in alignment between bisulfite modified and un-modified sequences. As a stringent quality filter, only reads with > 90% identity (excepting bisulfite modifiable sites) and > 90% coverage to the reference sequence were considered for further analysis. Pairwise alignments were used to construct reference sequenced anchored multiple read alignments by preserving alignment gaps in the sequence read but removing alignment gap-columns in the reference sequence. At each methylation informative site (C of the CpG in the unmodified template) the fraction of bisulfite non-conversion (Ccount/(Ccount + Tcount)) were scored across all reads. The mean of the methylation rate over the 19 informative sites provided a “site based estimate” (SBE) of methylation for each sample. As a complementary approach we also produced a “read based estimate” (RBE) of methylation by categorising each sequence read based on the fraction of bisulfite converted informative sites: methylated (< = 20%), indeterminate (> 20%, < 80%) or unmethylated (> = 80%). The RBE for the sample was calculated as the fraction methylated/(methylated + unmethylated) ignoring indeterminate classification reads. In these datasets the fraction of indeterminate reads was always < 1%. Sequence and alignment manipulation was implemented in Perl (version 5.10.1), quantization and analysis in R (version 3.0.0).

### Statistical analysis

Statistical analysis was performed using Graphpad Prism version 5.04 (GraphPad). All data sets showed a normal distribution, passing the Kolmogorov-Smirnov normality test at α = 0.05. One-way Analysis of Variance (ANOVA) comparing observed percent methylation values between different samples within the constructed panel was performed with the Tukey’s Multiple Comparison post-hoc test. Correlation analysis was performed using a two-way Pearson correlation.

## Electronic supplementary material

Additional file 1: **Shows the relative positions of primers and probes within the region of the p14** ^**ARF**^ **promoter used for bisulfite amplicon next-generation sequencing PCR.** (PPTX 52 KB)

Additional file 2: **Description of MethyLight PCR and primer/probe design.** (DOCX 21 KB)

Additional file 3: **Shows the linear regression analysis of the bisulfite amplicon NGS data.** (PPTX 166 KB)

Additional file 4: **Shows the predicted statistical error associated with dPCR measurements [** [55] **].** (XLSX 43 KB)

Additional file 5: **Shows the MIQE checklist for authors, reviewers and editors [** [40] **,** [56] **,** [57] **].** (DOCX 28 KB)

Additional file 6: **Shows a summary of primers and probes.** (DOCX 19 KB)

Additional file 7: **Shows the assay performance for qPCR in accordance with the MIQE guidelines.** (PPTX 2 MB)

Additional file 8: **Shows the Digital MIQE checklist for authors, reviewers and editors [** [40, 56] **-** [58] **].** (DOCX 25 KB)

Additional file 9: **Shows the assay performance for digital PCR in accordance with the digital MIQE guidelines.** (PPTX 469 KB)

## Abbreviations

MSRE: Methylation sensitive restriction enzyme
MDRE: Methylation dependent restriction enzyme
dPCR: Digital PCR
NGS: Next generation sequencing
RE: Restriction enzyme
MID: Mulitplex identifier.
