# Supplementary material for: Quantification of epigenetic biomarkers: an evaluation of established and emerging methods for DNA methylation analysis
Source: BMC Genomics. 2014 Dec 23;15(1):1174. doi: 10.1186/1471-2164-15-1174 (PMC4523014; doi:10.1186/1471-2164-15-1174)
Supplement: Supplementary file 1 — Additional file 1: Shows the relative positions of primers and probes within the region of the p14 ARF promoter used for bisulfite amplicon next-generation sequencing PCR. (PPTX 52 KB) [file 12864_2014_7081_MOESM1_ESM.pptx]

## Slide 1
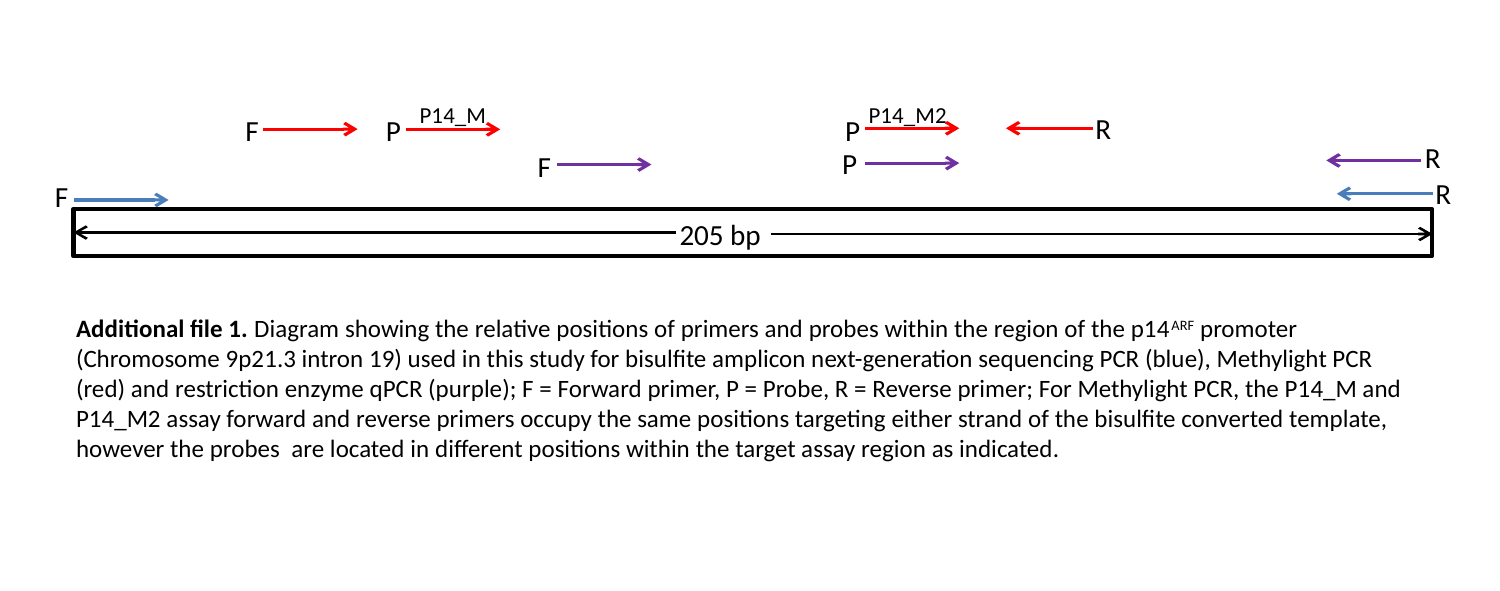

P14_M
P14_M2
R
F
P
P
R
P
F
R
F
205 bp
Additional file 1. Diagram showing the relative positions of primers and probes within the region of the p14ARF promoter (Chromosome 9p21.3 intron 19) used in this study for bisulfite amplicon next-generation sequencing PCR (blue), Methylight PCR (red) and restriction enzyme qPCR (purple); F = Forward primer, P = Probe, R = Reverse primer; For Methylight PCR, the P14_M and P14_M2 assay forward and reverse primers occupy the same positions targeting either strand of the bisulfite converted template, however the probes are located in different positions within the target assay region as indicated.
