# Supplementary material for: Quantification of epigenetic biomarkers: an evaluation of established and emerging methods for DNA methylation analysis
Source: BMC Genomics. 2014 Dec 23;15(1):1174. doi: 10.1186/1471-2164-15-1174 (PMC4523014; doi:10.1186/1471-2164-15-1174)
Supplement: Supplementary file 2 — Additional file 2: Description of MethyLight PCR and primer/probe design. (DOCX 21 KB) [file 12864_2014_7081_MOESM2_ESM.docx]

**Additional file 2.** Description of Methylight PCR and primer/probe design.

| Primer/probe | Sequence (5’-3’) |
| --- | --- |
| Forward (p14_M) | CGGGGACGCGAGTAGTATT |
| Reverse (p14_M) | GCCTAAAACGCAACTCCAAA |
| Probe (p14_M) | FAM-CGGGAGCGCGGTTGTTTTTG-BHQ |
| Forward (p14_M2) | CGAAAACGCGAACAACACC |
| Reverse (p14_M2) | GTTTGGAACGTAATTTTAGGTAGTTC |
| Probe (p14_M2) | HEX-CGAAAACGCGACTATTCCTA-BHQ |

3’-AAACCTCAACGCAAAATCCG-5’

Forward strand (P14_M) 5’-CGGGGACGCGAGTAGTATTAGAATTCGCGGGAGCGCGGTTGTTTTTGGTAGGGTCGTGTTAGGTGACGGATGTAGTTAGGGGGCGAGTTGTTTGGAGTTGCGTTTTAGGC–3’

Reverse strand (P14_M2) 3’-GCTTTTGCGCTTGTTGTGGTTTTAGGCGCTTTTGCGCTGATAAGGATTATTTTGGCATAGTTTATTGCTTATATTGATTTTTTGCTTGATGGATTTTAATGCAAGGTTTG-5’

5’-CGAAAACGCGAACAACACC-3’ 5’-CGAAAACGCGACTATTCCTA-3’

** ****** * * * *** **** * **** * ** * ** *** * * * *** ** ** ** *** * * * ** **** * *

**Primer and probe sequences for Methylight PCR p14 assays and alignment of forward and reverse strands of the p14 target region after bisulfite conversion**. The p14 assays are methylation specific, therefore the sequences shown have been bisulfite converted from the 100% methylated template. *indicates complementarity between forward and reverse strands. Cytosine nucleotides located at CpG sites are highlighted in blue to indicate methylation (not bisulfite converted). Thymine nucleotides highlighted in red have been bisulfite converted from non-methylated Cytosines (Cytosine nucleotides are bisulfite converted to Uracil but will be incorporated as Thymines in subsequent PCR cycles). The positions of the P14_M and P14_M2 primers and probes are shown with colour shading to indicate which assays they correspond to in the above table. BHQ = Black Hole Quencher.

Description of bisulfite PCR using the p14 Methylight assays

The P14_M and P14_M2 primers and probes were designed to amplify only the original forward or reverse strands of the p14 target region respectively. This diagram highlights why this strategy is required for Methylight PCR, due to the incomplementarity that exists between both strands after bisfulite conversion. To describe the bisulfite PCR process, for the P14_M assay for example, in the first PCR cycle of Methylight PCR, the reverse primer binds to the bisulfite converted forward strand and generates a complementary product; in the next cycle, the forward primer and probe will bind to the reverse strand generated after the first cycle but not to the starting reverse strand as the latter is not complementary to the starting forward strand. Therefore, the starting reverse strand should not be involved in PCR amplification by the p14_M assay. The alignment between the forward and reverse strands also highlights the extent of the incomplementarity that exists between both strands of bisulfite converted DNA, resulting in the DNA existing in a largely single-stranded conformation, as 47% of nucleotides in the 100% methylated template (above) are non-complementary and 68% in the 0% methylated template.
