# Supplementary material for: Quantification of epigenetic biomarkers: an evaluation of established and emerging methods for DNA methylation analysis
Source: BMC Genomics. 2014 Dec 23;15(1):1174. doi: 10.1186/1471-2164-15-1174 (PMC4523014; doi:10.1186/1471-2164-15-1174)
Supplement: Supplementary file 3 — Additional file 3: Shows the linear regression analysis of the bisulfite amplicon NGS data. (PPTX 166 KB) [file 12864_2014_7081_MOESM3_ESM.pptx]

## Slide 1
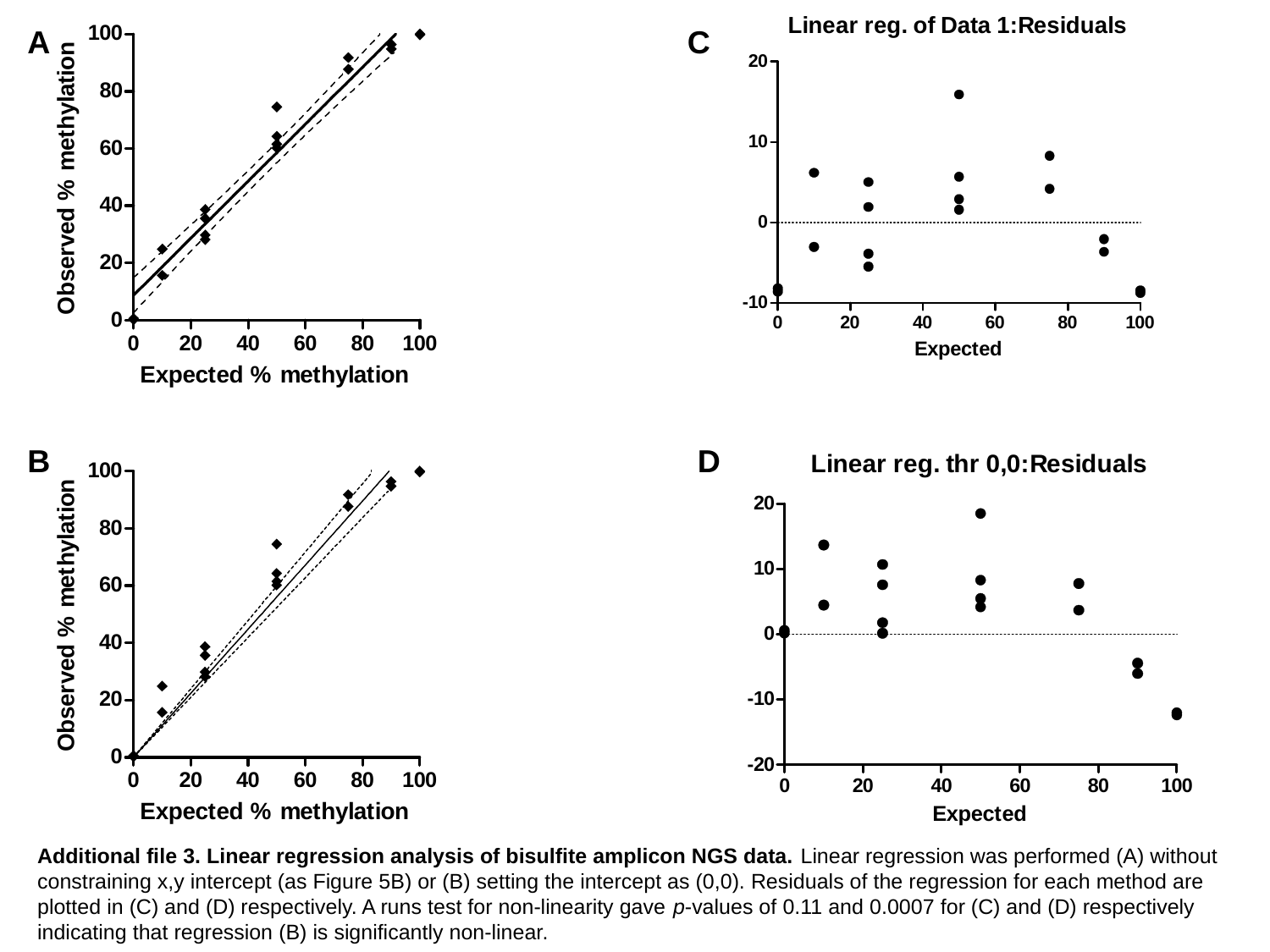

A
C
B
D
Additional file 3. Linear regression analysis of bisulfite amplicon NGS data. Linear regression was performed (A) without constraining x,y intercept (as Figure 5B) or (B) setting the intercept as (0,0). Residuals of the regression for each method are plotted in (C) and (D) respectively. A runs test for non-linearity gave p-values of 0.11 and 0.0007 for (C) and (D) respectively indicating that regression (B) is significantly non-linear.
