# Supplementary material for: Quantification of epigenetic biomarkers: an evaluation of established and emerging methods for DNA methylation analysis
Source: BMC Genomics. 2014 Dec 23;15(1):1174. doi: 10.1186/1471-2164-15-1174 (PMC4523014; doi:10.1186/1471-2164-15-1174)
Supplement: Supplementary file 5 — Additional file 5: Shows the MIQE checklist for authors, reviewers and editors [ [40] , [56] , [57] ]. (DOCX 28 KB) [file 12864_2014_7081_MOESM5_ESM.docx]

**Additional file 5. MIQE checklist for authors, reviewers and editors­**

| **ITEM TO CHECK** | **IMPORTANCE** | **CHECKLIST** | **COMMENTS/WHERE?** |
| --- | --- | --- | --- |
| **EXPERIMENTAL DESIGN** | | | |
| Definition of experimental and control  Groups | E | N/A | ­­ |
| Number within each group | E | N/A |  |
| Assay carried out by core lab or  Investigators’ lab? | D | YES | Investigators’ lab |
| Acknowledgement of authors'  contributions | D | YES | Authors’ contributions section |
| **SAMPLE** | | | |
| Description | E | YES | Materials and Methods |
| Volume/mass of sample processed | D | YES | Materials and Methods |
| Microdissection or macrodissection | E | N/A |  |
| Processing procedure | E | N/A |  |
| If frozen - how and how quickly? | E | N/A |  |
| If fixed - with what, how quickly? | E | N/A |  |
| Sample storage conditions and duration  (especially for FFPE samples) | E | YES | Materials and Methods |
| **NUCLEIC ACID EXTRACTION** | | | |
| Procedure and/or instrumentation | E | N/A | DNA supplied by manufacturer (materials and methods) |
| Name of kit and details of any  modifications | E | N/A |  |
| Source of additional reagents used | D | N/A |  |
| Details of DNase or RNAse treatment | E | N/A |  |
| Contamination assessment (DNA or  RNA) | E | N/A |  |
| Nucleic acid quantification | E | YES | Materials and Methods |
| Instrument and method | E | YES | Materials and Methods |
| Purity (A260/A280) | D | YES | Available on request |
| Yield | D | N/A |  |
| RNA integrity method/instrument | E | N/A |  |
| RIN/RQI or Cq of 3' and 5' transcripts | E | N/A |  |
| Electrophoresis traces | D | N/A |  |
| Inhibition testing (Cq dilutions, spike or other) | E | YES | Additional file 7 |
| **REVERSE TRANSCRIPTION** | | | |
| Complete reaction conditions | E | N/A |  |
| Amount of RNA and reaction volume | E | N/A |  |
| Priming oligonucleotide (if using GSP) and concentration | E | N/A |  |
| Reverse transcriptase and  concentration | E | N/A |  |
| Temperature and time | E | N/A |  |
| Manufacturer of reagents and  catalogue numbers | D | N/A |  |
| Cqs with and without RT | D | N/A |  |
| Storage conditions of cDNA | D | N/A |  |
| **qPCR TARGET INFORMATION** | | | |
| Sequence accession number | E | YES | CDKN2A (p14^ARF^): NC_000009.12  COL2A1: NC_000012.12 |
| Location of amplicon | D | YES | Additional file 6 |
| Amplicon length | E | YES | Additional file 6 |
| In silico specificity screen (BLAST,  etc) | E | YES | Available on request |
| Pseudogenes, retropseudogenes or  other homologs? | D | YES | None detected by BLASTn |
| Sequence alignment | D | YES | Available on request |
| Secondary structure analysis of  Amplicon | D | N/A |  |
| Location of each primer by exon or  intron (if applicable) | E | YES | Additional file 6 |
| What splice variants are targeted? | E | N/A |  |
| **qPCR OLIGONUCLEOTIDES** | | | |
| Primer sequences | E | YES | Additional file 6 |
| RTPrimerDB Identification Number | D | N/A |  |
| Probe sequences | D | YES | Additional file 6 |
| Location and identity of any  modifications | E | YES | Additional file 6 |
| Manufacturer of oligonucleotides | D | YES | Materials and Methods |
| Purification method | D | YES | HPLC |
| **qPCR PROTOCOL** | | | |
| Complete reaction conditions | E | YES | Materials and Methods |
| Reaction volume and amount of  cDNA/DNA | E | YES | Materials and Methods |
| Primer, (probe), Mg++ and dNTP  concentrations | E | YES | Materials and Methods; Manufacturer’s proprietary |
| Polymerase identity and concentration | E | YES | AmpliTaq Gold® DNA Polymerase; concentration is Manufacturer’s proprietary |
| Buffer/kit identity and manufacturer | E | YES | Materials and Methods |
| Exact chemical constitution of the  buffer | D | NO | Manufacturer’s proprietary |
| Additives (SYBR Green I, DMSO,  etc.) | E | N/A |  |
| Manufacturer of plates/tubes and catalog number | D | YES | Life Technologies 96-well plates (Cat. no. 4306737) |
| Complete thermocycling parameters | E | YES | Materials and Methods |
| Reaction setup (manual/robotic) | D | YES | Manual |
| Manufacturer of qPCR instrument | E | YES | Materials and Methods |
| **qPCR VALIDATION** | | | |
| Evidence of optimisation (from  gradients) | D | YES | Standard curves shown in Additional file 7 |
| Specificity (gel, sequence, melt, or  digest) | E | YES | Available on request |
| For SYBR Green I, Cq of the NTC | E | N/A |  |
| Standard curves with slope and y-intercept | E | YES | Additional file 7 |
| PCR efficiency calculated from slope | E | YES | Additional file 7 |
| Confidence interval for PCR efficiency or standard error | D | N/A |  |
| R^2^ of standard curve | E | YES | Additional file 7 |
| Linear dynamic range | E | YES | Additional file 7 |
| Cq variation at lower limit | E | YES | Additional file 7 |
| Confidence intervals throughout range | D | YES | Tables 1,2 and Figures 1,2 |
| Evidence for limit of detection | E | YES | Additional file 7 |
| If multiplex, efficiency and LOD of each assay. | E | N/A |  |
| **DATA ANALYSIS** | | | |
| qPCR analysis program (source, version) | E | YES | Materials and Methods |
| Cq method determination | E | YES | Materials and Methods |
| Outlier identification and disposition | E | YES | Table 1, Figure 1 and Results |
| Results of NTCs | E | YES | All NTCs showed no amplification (as shown in Additional file 7). |
| Justification of number and choice of  reference genes | E | YES | COL2A1 assay has been previously described as an effective methylation independent reference control gene for Methylight [40,56,57]. |
| Description of normalisation method | E | YES | Materials and Methods |
| Number and concordance of biological replicates | D | N/A |  |
| Number and stage (RT or qPCR) of  technical replicates | E | YES | Materials and Methods |
| Repeatability (intra-assay variation) | E | YES | Additional file 7 |
| Reproducibility (inter-assay variation,  %CV) | D | YES | Standard deviation measurements in Results |
| Power analysis | D | N/A |  |
| Statistical methods for result significance | E | YES | Materials and Methods and Results |
| Software (source, version) | E | YES | Materials and Methods |
| Cq or raw data submission using RDML | D | N/A |  |
| E = Essential; D = Desirable | | | |
