# Supplementary material for: Quantification of epigenetic biomarkers: an evaluation of established and emerging methods for DNA methylation analysis
Source: BMC Genomics. 2014 Dec 23;15(1):1174. doi: 10.1186/1471-2164-15-1174 (PMC4523014; doi:10.1186/1471-2164-15-1174)
Supplement: Supplementary file 6 — Additional file 6: Shows a summary of primers and probes. (DOCX 19 KB) [file 12864_2014_7081_MOESM6_ESM.docx]

| Gene target | Application | Primer/Probe | PCR method | Sequence (5’- 3’) | Amplicon length (bp) | Genomic location |
| --- | --- | --- | --- | --- | --- | --- |
| P14 | Methylight | Forward (F, p14_M) | qPCR, dPCR | CGGGGACGCGAGTAGTATT | 110 | Chromosome 9p21.3 (gene promoter) |
|  |  | Reverse (F, p14_M) | qPCR, dPCR | GCCTAAAACGCAACTCCAAA |  |  |
|  |  | Probe (F, p14_M) | qPCR, dPCR | FAM-CGGGAGCGCGGTTGTTTTTG-BHQ |  |  |
|  |  | Forward (R, p14_M2) | dPCR | CGAAAACGCGAACAACACC |  |  |
|  |  | Reverse (R, p14_M2) | dPCR | GTTTGGAACGTAATTTTAGGTAGTTC |  |  |
|  |  | Probe (R, p14_M2) | dPCR | HEX-CGAAAACGCGACTATTCCTA-BHQ |  |  |
| COL2A1 | Methylight | Forward | qPCR, dPCR | TCTAACAATTATAAACTCCAACCACCAA | 92 | Chromosome 12q13.11 (intron 19) |
|  |  | Reverse | qPCR, dPCR | GGGAAGATGGGATAGAAGGGAATAT |  |  |
|  |  | Probe | qPCR, dPCR | FAM-CCTTCATTCTAACCCAATACCTATCCCACCTCTAAA-BHQ |  |  |
| P14 | Restriction enzyme qPCR | Forward | qPCR, dPCR | CGTGTCAGGTGACGGATGTA | 111 | Chromosome 9p21.3 (gene promoter) |
|  |  | Reverse | qPCR, dPCR | ACCATCTTCCCACCCTCAG |  |  |
|  |  | Probe | qPCR, dPCR | FAM-AGTTGCGTTCCAGGCGTCCG-BHQ |  |  |
| P14 | Bisulfite amplicon NGS | Forward MID 1 | BS PCR | **CGTATCGCCTCCCTCGCGCCATCAGACGAGTGCGTGAGGGGAGTTAGGAATAAAATAA** | 265 | Chromosome 9p21.3 (gene promoter) |
|  |  | Forward MID 2 | BS PCR | **CGTATCGCCTCCCTCGCGCCATCAGACGCTCGACAGAGGGGAGTTAGGAATAAAATAA** |  |  |
|  |  | Forward MID 3 | BS PCR | **CGTATCGCCTCCCTCGCGCCATCAGAGACGCACTCGAGGGGAGTTAGGAATAAAATAA** |  |  |
|  |  | Forward MID 4 | BS PCR | **CGTATCGCCTCCCTCGCGCCATCAGAGCACTGTAGGAGGGGAGTTAGGAATAAAATAA** |  |  |
|  |  | Forward MID 5 | BS PCR | **CGTATCGCCTCCCTCGCGCCATCAGATCAGACACGGAGGGGAGTTAGGAATAAAATAA** |  |  |
|  |  | Forward MID 6 | BS PCR | **CGTATCGCCTCCCTCGCGCCATCAGATATCGCGAGGAGGGGAGTTAGGAATAAAATAA** |  |  |
|  |  | Forward MID 7 | BS PCR | **CGTATCGCCTCCCTCGCGCCATCAGCGTGTCTCTAGAGGGGAGTTAGGAATAAAATAA** |  |  |
|  |  | Forward MID 8 | BS PCR | **CGTATCGCCTCCCTCGCGCCATCAGCTCGCGTGTCGAGGGGAGTTAGGAATAAAATAA** |  |  |
|  |  | Forward MID 9 | BS PCR | **CGTATCGCCTCCCTCGCGCCATCAGTAGTATCAGCGAGGGGAGTTAGGAATAAAATAA** |  |  |
|  |  | Forward MID 10 | BS PCR | **CGTATCGCCTCCCTCGCGCCATCAGTCTCTATGCGGAGGGGAGTTAGGAATAAAATAA** |  |  |
|  |  | Reverse | BS PCR | **CTATGCGCCTTGCCAGCCCGCTCAGACCACCATCTTCCCACCCTCAA** |  |  |

**Additional file 6.** For Methylight PCR analysis of P14, primers and probes were designed that only amplify either the starting forward (referred to as P14_M in manuscript) or reverse strands (referred to as P14_M2 in manuscript) as indicated (F = Forward strand; R = Reverse strand); For Bisulfite amplicon NGS, fusion primers contained the Roche adapter sequence (blue), key sequence (red), Multiplex Identifier (MID) (orange, forward primer only) and gene-specific sequence in green; the same reverse primer was used with each MID fusion forward primer; BS = Bisulfite, BHQ = Black Hole Quencher.
