# Supplementary material for: Quantification of epigenetic biomarkers: an evaluation of established and emerging methods for DNA methylation analysis
Source: BMC Genomics. 2014 Dec 23;15(1):1174. doi: 10.1186/1471-2164-15-1174 (PMC4523014; doi:10.1186/1471-2164-15-1174)
Supplement: Supplementary file 7 — Additional file 7: Shows the assay performance for qPCR in accordance with the MIQE guidelines. (PPTX 2 MB) [file 12864_2014_7081_MOESM7_ESM.pptx]

## Slide 1
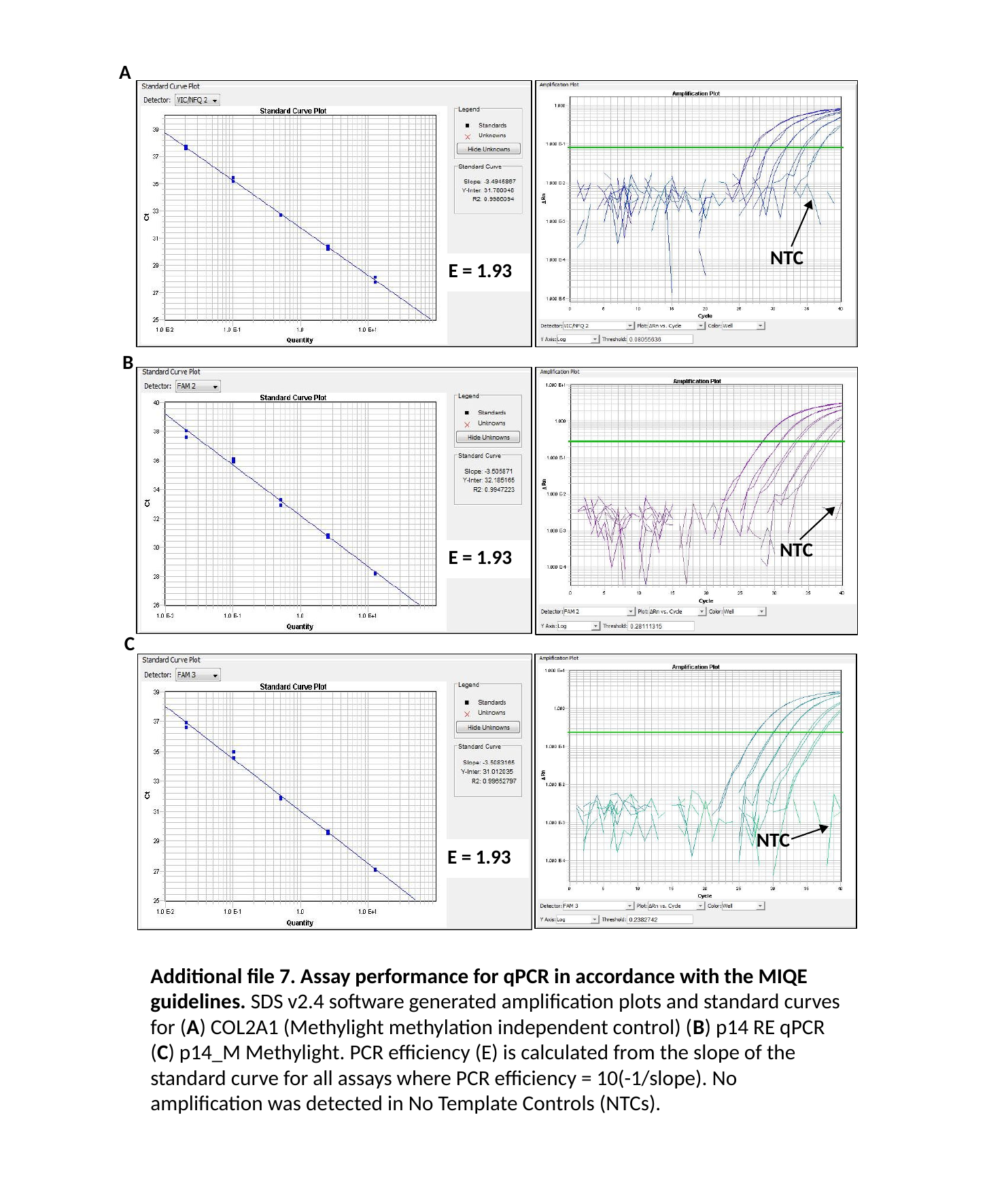

Additional file 7. Assay performance for qPCR in accordance with the MIQE guidelines. SDS v2.4 software generated amplification plots and standard curves for (A) COL2A1 (Methylight methylation independent control) (B) p14 RE qPCR (C) p14_M Methylight. PCR efficiency (E) is calculated from the slope of the standard curve for all assays where PCR efficiency = 10(-1/slope). No amplification was detected in No Template Controls (NTCs).
