# Supplementary material for: Quantification of epigenetic biomarkers: an evaluation of established and emerging methods for DNA methylation analysis
Source: BMC Genomics. 2014 Dec 23;15(1):1174. doi: 10.1186/1471-2164-15-1174 (PMC4523014; doi:10.1186/1471-2164-15-1174)
Supplement: Supplementary file 8 — Additional file 8: Shows the Digital MIQE checklist for authors, reviewers and editors [ [40, 56] - [58] ]. (DOCX 25 KB) [file 12864_2014_7081_MOESM8_ESM.docx]

**Additional file 8. Digital MIQE checklist for authors, reviewers and editors**

| **ITEM TO CHECK** | **IMPORTANCE** | **CHECKLIST** | **COMMENTS/WHERE?** |
| --- | --- | --- | --- |
| **EXPERIMENTAL DESIGN** | | | |
| Definition of experimental and control  Groups | E | N/A |  |
| Number within each group. | E | N/A |  |
| Assay carried out by core lab or  Investigators’ lab? | D | YES | Investigators’ lab |
| Power analysis | D | N/A |  |
| **SAMPLE** | | | |
| Description | E | YES | Materials and Methods |
| Volume or mass of sample processed | E | YES | Materials and Methods |
| Microdissection or macrodissection | E | N/A |  |
| Processing procedure | E | N/A |  |
| If frozen - how and how quickly? | E | N/A |  |
| If fixed - with what, how quickly? | E | N/A |  |
| Sample storage conditions and duration (especially for FFPE samples) | E | YES | Materials and Methods |
| **NUCLEIC ACID EXTRACTION** |  |  |  |
| Quantification - instrument/method | E | N/A | DNA supplied by manufacturer (materials and methods) |
| Storage conditions: temperature, concentration, duration, buffer | E | YES | Materials and Methods |
| DNA or RNA quantification | E | YES | DNA |
| Quality/integrity-instrument/method; e.g. RIN/RQI and trace or 3’:5’ | E | N/A |  |
| Template structural information | E | N/A |  |
| Template modification (digestion, sonication, pre-amplification etc.) | E | YES | Materials and Methods |
| Template treatment (initial heating or chemical denaturation) | E | YES | Materials and Methods |
| Inhibition dilution or spike | E | YES | DNA samples were diluted after RE digestion and bisulfite treatment as described in Materials and Methods in order to reduce effects of potential inhibitors |
| DNA contamination assessment of RNA sample | E | N/A |  |
| Details of DNase treatment where performed | E | N/A |  |
| Manufacturer of reagents used and catalogue number | D | N/A |  |
| Storage of nucleic acid: temperature, concentration, duration, buffer | E | YES | Materials and Methods |
| **REVERSE TRANSCRIPTION (If necessary)** |  |  |  |
| cDNA priming method + concentration | E | N/A |  |
| One or two step protocol | E | N/A |  |
| Amount of RNA used per reaction | E | N/A |  |
| Detailed reaction components and conditions | E | N/A |  |
| RT efficiency | D | N/A |  |
| Estimated copies measured with and without addition of RT* | D | N/A |  |
| Manufacturer of reagents used and catalogue number | D | N/A |  |
| Reaction volume (for two step reverse transcription reaction) | D | N/A |  |
| Storage of cDNA: temperature, concentration, duration, buffer | D | N/A |  |
| **dPCR TARGET INFORMATION** |  |  |  |
| Sequence accession number | E | YES | CDKN2A (p14^ARF^): NC_000009.12  COL2A1: NC_000012.12 |
| Location of amplicon | D | YES | Additional file 6 |
| Amplicon length | E | YES | Additional file 6 |
| In silico specificity screen (BLAST, etc) | E | YES | Available on request |
| Pseudogenes, retropseudogenes or other homologs? | D | YES | None detected by BLASTn |
| Sequence alignment | D | YES | Available on request |
| Secondary structure analysis of amplicon and GC content | D | N/A |  |
| Location of each primer by exon or intron (if applicable) | E | YES | Additional file 6 |
| Where appropriate, which splice variants are targeted? | E | N/A |  |
| **dPCR OLIGONUCLEOTIDES** |  |  |  |
| Primer sequences and/or amplicon context sequence** | E | YES | Additional file 6 |
| RTPrimerDB Identification Number | D | N/A |  |
| Probe sequences** | D | YES | Additional file 6 |
| Location and identity of any modifications | E | YES | Additional file 6 |
| Manufacturer of oligonucleotides | D | YES | Materials and Methods |
| Purification method | D | YES | HPLC |
| **dPCR PROTOCOL** |  |  |  |
| Complete reaction conditions | E | YES | Materials and Methods |
| Reaction volume and amount of RNA/cDNA/DNA | E | YES | Materials and Methods |
| Primer, (probe), Mg++ and dNTP concentrations | E | YES | Materials and Methods; Manufacturer’s proprietary |
| Polymerase identity and concentration | E | YES | AmpliTaq Gold® DNA Polymerase; concentration is Manufacturer’s proprietary |
| Buffer/kit Catalogue No and manufacturer | E | YES | Materials and Methods |
| Exact chemical constitution of the buffer | D | NO | Manufacturers’ proprietary |
| Additives (SYBR Green I, DMSO, etc.) | E | N/A |  |
| Plates/tubes Catalogue No and manufacturer | D | YES | Materials and Methods |
| Complete thermocycling parameters | E | YES | Materials and Methods |
| Reaction setup | D | YES | Materials and Methods |
| Gravimetric or volumetric dilutions (manual/robotic) | D | YES | Manual |
| Total PCR reaction volume prepared | D | YES | Materials and Methods |
| Partition number | E | YES | Materials and Methods |
| Individual partition volume | E | YES | 0.85 nL |
| Total volume of the partitions measured (effective reaction size) | E | YES | 770 partitions (digital Methylight)  3080 partitions (MDRE/MSRE dPCR) |
| Partition volume variance/standard deviation | D | N/A |  |
| Comprehensive details and appropriate use of controls | E | YES | Materials and Methods and Results |
| Manufacturer of dPCR instrument | E | YES | Materials and Methods |
| **dPCR VALIDATION** |  |  |  |
| Optimisation data for the assay | D | YES | Available on request |
| Specificity (when measuring rare mutations, pathogen sequences etc.) | E | N/A |  |
| Limit of detection of calibration control | D | N/A |  |
| If multiplexing, comparison with singleplex assays | E | YES | Results and Discussion, Additional file 9 |
| **DATA ANALYSIS** |  |  |  |
| Average copies per partition (λ or equivalent ) | E | YES | Results, Additional file 4 |
| dPCR analysis program (source, version) | E | YES | Materials and Methods |
| Outlier identification and disposition | E | N/A |  |
| Results of NTCs | E | YES | All NTCs gave negative results as shown in Additional file 9 |
| Examples of positive(s) and negative experimental results as supplemental data | E | YES | Additional file 9 |
| Where appropriate, justification of number and choice of reference genes | E | YES | COL2A1 assay has been previously described as an effective methylation independent reference control gene for Methylight [40,56,57]. |
| Where appropriate, description of normalisation method | E | YES | Materials and Methods |
| Number and concordance of biological replicates | D | N/A |  |
| Number and stage (RT or qPCR) of technical replicates | E | YES | Materials and Methods |
| Repeatability (intra-assay variation) | E | N/A | See below reproducibility analysis |
| Reproducibility (inter-assay/user/lab etc. variation ) | D | YES | Standard deviation measurements in Results |
| Experimental variance or confidence interval*** | E | YES | Results and Additional file 4 |
| Statistical methods used for analysis | E | YES | Materials and Methods and Results |
| Data submission using RDML | D | N/A |  |

All essential information (E) must be submitted with the manuscript. Desirable information (D) should be submitted if possible.

* Assessing the absence of DNA using a no RT assay (or where RT has been inactivated) is essential when first extracting RNA. Once the sample has been validated as DNA-free, inclusion of a no-RT control is desirable, but no longer essential.

** Disclosure of the primer and probe sequence is highly desirable and strongly encouraged. However, since not all commercial pre-designed assay vendors provide this information when it is not available assay context sequences must be submitted [58].

*** When single dPCR experiments are performed, the variation due to counting error alone should be calculated from the binomial (or suitable equivalent) distribution.
