# Supplementary material for: Quantification of epigenetic biomarkers: an evaluation of established and emerging methods for DNA methylation analysis
Source: BMC Genomics. 2014 Dec 23;15(1):1174. doi: 10.1186/1471-2164-15-1174 (PMC4523014; doi:10.1186/1471-2164-15-1174)
Supplement: Supplementary file 9 — Additional file 9: Shows the assay performance for digital PCR in accordance with the digital MIQE guidelines. (PPTX 469 KB) [file 12864_2014_7081_MOESM9_ESM.pptx]

## Slide 1
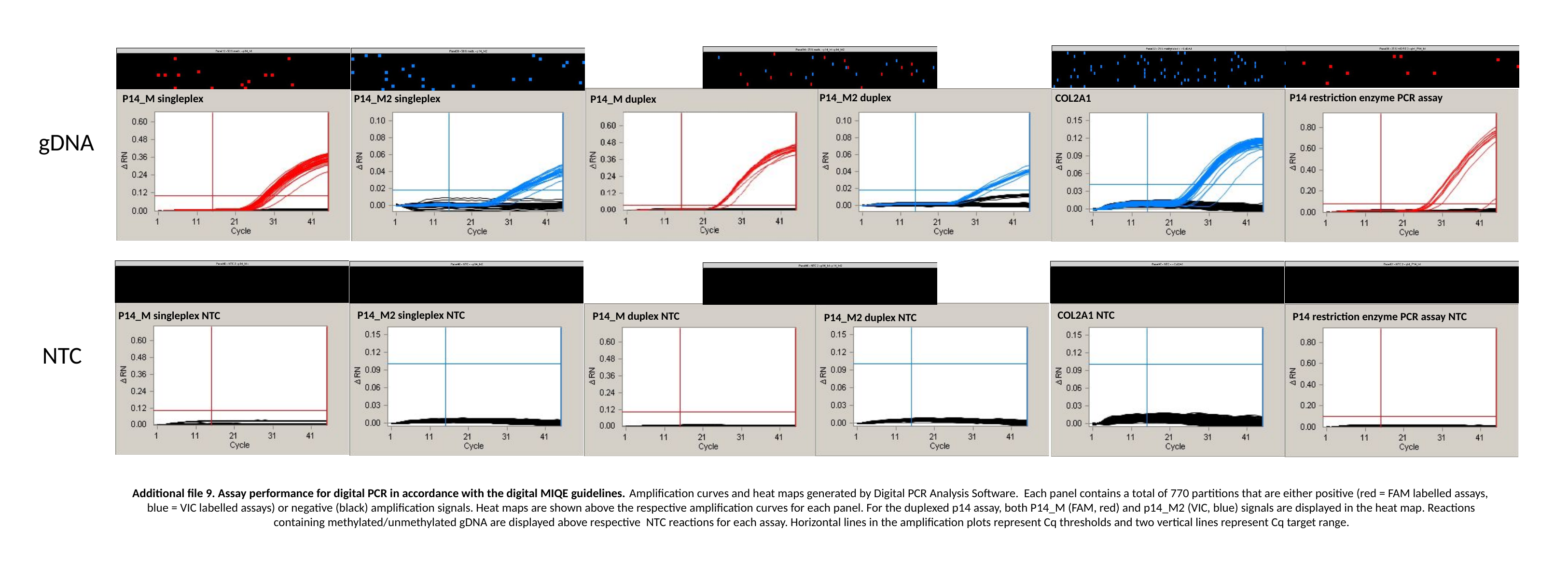

P14 restriction enzyme PCR assay
P14_M2 duplex
COL2A1
P14_M2 singleplex
P14_M singleplex
P14_M duplex
COL2A1 NTC
P14_M2 singleplex NTC
P14_M singleplex NTC
P14_M duplex NTC
P14 restriction enzyme PCR assay NTC
P14_M2 duplex NTC
gDNA
NTC
Additional file 9. Assay performance for digital PCR in accordance with the digital MIQE guidelines. Amplification curves and heat maps generated by Digital PCR Analysis Software. Each panel contains a total of 770 partitions that are either positive (red = FAM labelled assays, blue = VIC labelled assays) or negative (black) amplification signals. Heat maps are shown above the respective amplification curves for each panel. For the duplexed p14 assay, both P14_M (FAM, red) and p14_M2 (VIC, blue) signals are displayed in the heat map. Reactions containing methylated/unmethylated gDNA are displayed above respective NTC reactions for each assay. Horizontal lines in the amplification plots represent Cq thresholds and two vertical lines represent Cq target range.
